# Complex Impedance and Modulus Analysis on Porous and Non-Porous Scaffold Composites Due to Effect of Hydroxyapatite/Starch Proportion

**DOI:** 10.3390/polym15020320

**Published:** 2023-01-08

**Authors:** Chong You Beh, Ee Meng Cheng, Xiao Jian Tan, Nashrul Fazli Mohd Nasir, Mohd Shukry Abdul Majid, Mohd Ridzuan Mohd Jamir, Shing Fhan Khor, Kim Yee Lee, Che Wan Sharifah Robiah Mohamad

**Affiliations:** 1 Department of Engineering and Built Environment, Tunku Abdul Rahman University of Management and Technology, Penang Branch, Pulau Pinang 11200, Malaysia; 2 Faculty of Electronic Engineering & Technology, Universiti Malaysia Perlis (UniMAP), Kangar 02600, Malaysia; 3 Advanced Communication Engineering (ACE) Centre of Excellence, Universiti Malaysia Perlis (UniMAP), Kangar 02600, Malaysia; 4 Centre for Multimodal Signal Processing, Tunku Abdul Rahman University of Management and Technology (TAR UMT), Jalan Genting Kelang, Setapak, Kuala Lumpur 53300, Malaysia; 5 Department of Electrical and Electronics Engineering, Faculty of Engineering and Technology, Tunku Abdul Rahman University of Management and Technology (TAR UMT), Jalan Genting Kelang, Setapak, Kuala Lumpur 53300, Malaysia; 6 Sports Engineering Research Centre (SERC), Universiti Malaysia Perlis (UniMAP), Kangar 02600, Malaysia; 7 Faculty of Mechanical Engineering & Technology, Universiti Malaysia Perlis (UniMAP), Kangar 02600, Malaysia; 8 Faculty of Electrical Engineering & Technology, Universiti Malaysia Perlis (UniMAP), Kangar 02600, Malaysia; 9 Lee Kong Chian Faculty of Engineering & Science, Sungai Long Campus, Jalan Sungai Long, Tunku Abdul Rahman University, Kajang, Cheras, Sungai Long City 43000, Malaysia

**Keywords:** hydroxyapatite, starch, porous composite, complex electric modulus, complex electric impedance

## Abstract

This study aims to investigate the electric responses (complex modulus and complex impedance analysis) of hydroxyapatite/starch bone scaffold as a function of hydroxyapatite/starch proportion and the microstructural features. Hence, the non-porous and porous hydroxyapatite/starch composites were fabricated with various hydroxyapatite/starch proportions (70/30, 60/40, 50/50, 40/60, 30/70, 20/80, and 10/90 wt/wt%). Microstructural analysis of the porous hydroxyapatite/starch composites was carried out by using scanning electron microscopy. It shows that the formation of hierarchical porous microstructures with high porosity is more significant at a high starch proportion. The complex modulus and complex impedance analysis were conducted to investigate the electrical conduction mechanism of the hydroxyapatite/starch composites via dielectric spectroscopy within a frequency range from 5 MHz to 12 GHz. The electrical responses of the hydroxyapatite/starch composites are highly dependent on the frequency, material proportion, and microstructures. High starch proportion and highly porous hierarchical microstructures enhance the electrical responses of the hydroxyapatite/starch composite. The material proportion and microstructure features of the hydroxyapatite/starch composites can be indirectly reflected by the simulated electrical parameters of the equivalent electrical circuit models.

## 1. Introduction

Carbohydrate polymers attract great attention from the industrial and academic communities due to their unique physicochemical properties, superior biocompatibility, ease of processability, environmental friendliness, and low cost [1,2,3]. The introduction of nano-sized active inorganic materials (metal, ceramic, silica, and glass) into polymeric materials (natural or synthetic polymer) to enhance the physicochemical performance of composites has gained considerable interest in electronic and biomedical applications [2,3,4,5,6]. Composites consist of two or more constituent materials with distinctive properties in an appropriate proportion. It has more favourable properties than conventional individual materials [1,3,5]. Hydroxyapatite nanoparticles have been widely used as an inorganic constituent material of composites in a variety of applications, especially in biomedical applications. This is due to its excellent biological performances, as well as its unique electrical properties [2,7,8]. However, the pure hydroxyapatite material exhibits a brittle nature [4,7]. According to the adhesive properties of carbohydrate polymers, the addition of starch in hydroxyapatite is used to ensure the intermolecular interactions between the hydroxyapatite nanoparticles and the starch matrix. It leads to the formation of effective composite interfaces. It facilitates the formation of stable porous microstructures [2,7,9]. Thus, the significant material proportion plays an important role in the material interactions of composite. The material interfacial surfaces of the porous composites with the electrical response enhancement can facilitate cellular activities for the improvement of their biological performance. It is important in the early stage of bone growth and remodeling [1,2,8].

In a heterogeneous system, the frequency-dependent complex modulus and complex impedance formalisms are usually used to explore the dynamics of mobile/bound charge carriers in bulk/interfacial regions of the composites [5,10,11,12]. Complex impedance emphasizes the grain boundary conduction process and highlights the large resistance. Complex modulus focuses on the electrical transport phenomena and small capacitance [4,13,14]. The variations of grain, grain boundary, interface, porosity, material proportion, and filler dispersion in the porous composites can be reflected in the complex modulus and complex impedance responses [4,10,12,15,16]. Many studies examined the electrical performances (complex modulus and complex impedance) of inorganic/organic composites for a variety of applications [1,4,13,17,18]. However, there are few literature reports on the study of the electrical responses (complex modulus and complex impedance) of ceramic/polymer composites, especially the investigation of the electric properties of porous ceramic/polymer composites according to their material proportion and microstructural feature. Investigating the effects of material proportions and microstructural features on electrical behaviour helps to assess the potential of complex modulus and complex impedance analysis as a non-destructive technique for evaluating the performance of porous ceramic/polymer composites.

Different analytical methods based on physical, chemical, and morphological evaluations have been applied to evaluate the composite characteristics, which are costly, time-consuming, and require skilled personnel. For this reason, developing low-cost, rapid, non-destructive, and user-friendly control techniques that could be applied at the initial stage of product development would be highly fascinating. This electrical study provides a better understanding of the effects of material proportions and microstructural features on the electrical responses of composites. This electrical assessment facilitates rapid product design and manufacturing of composite, especially bone tissue engineering. In this work, the non-porous and porous hydroxyapatite/starch composites with different hydroxyapatite/starch proportions (70/30, 60/40, 50/50, 40/60, 30/70, 20/80, and 10/90 wt/wt%) were prepared. Microstructural study of the hydroxyapatite/starch composites with various hydroxyapatite/starch proportions was conducted using scanning electron microscopy and porosity measurement. Complex modulus and complex impedance of the non-porous and porous hydroxyapatite/starch composites are investigated using dielectric spectroscopy within a frequency range from 5 MHz to 12 GHz. Equivalent electrical circuits were also developed based on the impedance of non-porous and porous hydroxyapatite/starch composites.

## 2. Materials and Methods

### 2.1. Preparation of Composites

The solvent casting/particulate leaching technique was implemented for the porous hydroxyapatite/starch composites. Hydroxyapatite (HAp) nanoparticles (CAS No. 1306-06-5, 20 nm; Sigma-Aldrich, St. Louis, MO, USA) and commercial corn starch (ST) (Siem Trading Pte Ltd, Singapore) were used to fabricate the porous ceramic/polymer composites. The porous composites with seven weight percentages of HAp/ST (70/30, 60/40, 50/50, 40/60, 30/70, 20/80, and 10/90 wt/wt%) were fabricated. ST was dispersed in distilled water at a starch:water ratio of 1:3 (*w*/*v*). Then, it was pregelatinized (45–65 °C) for ~1 h. Next, the HAp was added to the pregelatinized ST solution. The entire mixture was heated in a boiling water bath by stirring continuously until the gelatinization process was completed. Commercial particulate porogen (sodium chloride) was sieved using a U.S standard N0.50 sieve (0.297 mm mesh size) before being mixed uniformly into the mixture. The ratio of the weight of particulate porogen to the mixture was determined at a constant ratio., i.e., 2:1. The mixture was casted into the Teflon mould (diameter = 20 mm and height = 10 mm) and cooled (2–10 °C) for 2 h. The composite was heated again (110–140 °C) for 3 h after dehydration (80–90 °C) for 12 h. The particulate porogen of the composite was removed via the particulate leaching process using distilled water (distilled water was refreshed twice every 6 h for at least 24 h) for the formation of pores in the composite. The porous HAp/ST composites were dried (85–95 °C) after immersion in 95% of ethanol (Sigma-Aldrich, St. Louis, MO, USA). All porous HAp/ST composites were ground into a fine powder and pressed into the non-porous HAp/ST composites (diameter = 20 mm and height = 10 mm) using a uniaxial hydraulic press under 25 MPa pressure.

### 2.2. Composite Characterization

Each porous HAp/ST composite was cut through the cross-section area and the porous microstructure of the composite sample was evaluated by a Hitachi TM3000 scanning electron microscope (SEM) equipment (Ramsey, New Jersey, US). The cross-sectional areas of the sliced porous composite samples were coated with a very thin layer of conductive gold (Au) layer. The cross-sectional areas for each sample were measured using SEM with various magnifications (50×, 500×, and 3000×) at 15-kV observation mode. The porosity of the porous HAp/ST composites was evaluated using a liquid displacement method where ethanol was used as a liquid medium. It is described in Equtaion (1):

(1) $$\mathrm{Porosity}=\frac{W_{2}-W_{1}}{W_{2}-W_{3}}\times 100\%$$

where W_1_ is the dry weight, W_2_ is the soaked weight, and W_3_ is the suspended weight of composites. The measurement of each sample was performed in triplicates and averages were recorded. The porosity of the porous HAp/ST composites (average value ± standard deviation) is listed in Table 1. The dielectric measurement of the non-porous and porous HAp/ST composites was conducted via dielectric spectroscopy by using Keysight E5071C vector network analyser (Colorado Springs, CO 80907-3423 US) over a broadband frequency range from 5 MHz to 12 GHz). Keysight 85070E dielectric probe (Colorado Springs, CO 80907-3423, US) was equipped to measure the dielectric parameters after the calibration. The calibration was conducted using air, short circuit, and water at room temperature. The dielectric parameters are the real part (ε′) and imaginary part (ε″) of the complex dielectric permittivity (ε*), as shown in Equation (2):

(2) $$\varepsilon^{*}=\varepsilon^{\prime }-j\varepsilon^{\prime \prime }$$

where $j=\sqrt{-1}$. Meanwhile, the real part (M′) and imaginary part (M″) of the complex modulus $\left(M^{*}=\frac{1}{\varepsilon^{*}}\right)$ for the HAp/ST composites were calculated via Equations (3)–(5):

(3) $$M^{*}=M^{\prime }+jM^{\prime \prime }$$

(4) $$M^{\prime }=\frac{\varepsilon^{\prime }}{{\varepsilon^{\prime }}^{2}+{\varepsilon^{\prime \prime }}^{2}}$$

(5) $$M^{\prime \prime }=\frac{\varepsilon^{\prime \prime }}{{\varepsilon^{\prime }}^{2}+{\varepsilon^{\prime \prime }}^{2}}$$

The real part (Z′) and imaginary part (Z″) of the complex impedance (Z*) of the HAp/ST composites can be calculated via Equations (6)–(9):

(6) $$Z^{*}=Z^{\prime }+jZ^{\prime \prime }$$

(7) $$Z^{\prime }=\frac{1}{2\pi fC_{o}}\left[\frac{\varepsilon^{\prime \prime }}{{\varepsilon^{\prime }}^{2}+{\varepsilon^{\prime \prime }}^{2}}\right]$$

(8) $$Z^{\prime \prime }=\frac{1}{2\pi fC_{o}}\left[\frac{\varepsilon^{\prime }}{{\varepsilon^{\prime }}^{2}+{\varepsilon^{\prime \prime }}^{2}}\right]$$

(9) $$C_{o}=\frac{\varepsilon_{o}A}{d}$$

where ƒ is the frequency, ε_o_ is the free space permittivity (8.85 × 10^−12^ F/m), A is the sample area, and d is the sample thickness [16,18,19,20]. The magnitude of complex impedance (|Z*|) can be calculated by using Equation (10):

(10) $$\left|Z^{*}\right|=\sqrt{{Z^{\prime }}^{2}+{Z^{\prime \prime }}^{2}}$$

Equivalent electrical circuit models were simulated and fitted using the complex impedance and the ZView software version 2.9 (Ametek Scientific Instruments, Oak Ridge, TN 37830 US), respectively. The errors of |Z*| between the simulation (equivalent electrical circuit model) and measurement were examined using the Chi-squared (χ2) criteria.

## 3. Results and Discussion

**Figure 1:**
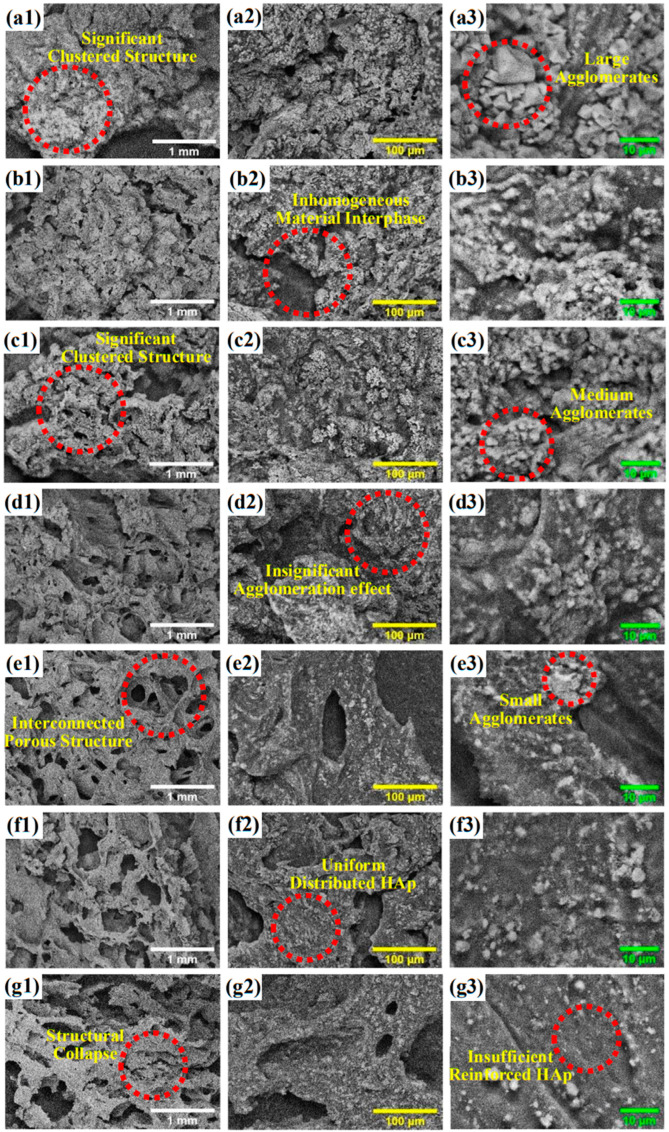
Morphological features of synthesized porous composites: ST30 (**a1**–**a3**), ST40 (**b1**–**b3**), ST50 (**c1**–**c3**), ST60 (**d1**–**d3**), ST70 (**e1**–**e3**), ST80 (**f1**–**f3**), and ST90 (**g1**–**g3**).

The surface morphology of the porous HAp/ST composites was determined by SEM equipment. Figure 1 shows the SEM images of the porous composites fabricated using 70/30 wt/wt% (ST30), 60/40 wt/wt% (ST40), 50/50 wt/wt% (ST50), 40/60 wt/wt% (ST60), 30/70 wt/wt% (ST70), 20/80 wt/wt% (ST80), and 10/90 wt/wt% (ST90) of HAp/ST proportions. The porosities of the porous HAp/ST composites with various HAp/ST proportions are shown in Table 1.

In Figure 1, the microstructures of the porous HAp/ST composites are highly varied due to the variation of the HAp/ST. It is owing to the occurrence of significant HAp/ST interactions. It is shown that a high amount of ST content leads to the formation of a more significant porous microstructure with high interconnectivity in the composite. It can be noticed in Figure 1. It could be seen that the clustered porous microstructures appear as pores distributed over the surface of the porous HAp/ST composites (30–50 wt% ST proportion). The porous HAp/ST composites exhibit less pronounced clustered porous microstructures when the proportions of ST are increased from 30 wt% to 50 wt%, as shown in Figure 1(a1–c1). The porosity of the porous HAp/ST composites increases from 62.22 ± 0.43 % (porous ST30 composite) to 66.36 ± 0.78% (porous ST50 composite), as shown in Table 1. As can be observed from Figure 1(a2–c2, a3–c3), the agglomeration effects and the material inhomogeneity can be seen in the porous HAp/ST composites due to the saturated HAp nanoparticles with high surface area to volume ratio. The large surface area causes more gathering of HAp nanoparticles in the ST matrix resulting in the formation of brittle ceramic phases [19,20,21].

Another form of microstructures in the porous HAp/ST composites with hierarchical porous morphology (interconnected porous microstructure) is exhibited when the ST proportion further increases. In Table 1, the porosity of the porous ST60 composite with 60 wt% ST proportion is 64.72 ± 0.44 %. In Figure 1(d1–d3), it can be seen that the agglomeration effect of the HAp nanoparticles is significantly reduced as the ST proportion dominates the porous HAp/ST composite. The high ST proportion causes a significant penetration of the ST matrix into the interparticle spaces and results in an efficient dispersion of HAp nanoparticles [21,22]. When the ST proportion was increased from 60 wt% to 70 wt%, the porosity of the porous HAp/ST composite increased (65.53 ± 0.67 %) with thick pore walls, as observed in Figure 1(e1,e2). Minor agglomeration of HAp nanoparticles still can be seen in the surface morphology of the porous ST70 composite as shown in Figure 1(e2,e3). The hierarchical porous microstructures are more remarkable in the porous HAp/ST composites when the ST proportion increases to 80 wt% and 90 wt%, as shown in Figure 1(f1-g1). The porous composite with 80 wt% of ST proportion (ST80) shows the highest porosity (66.56 ± 0.54 %). The high magnification (500× and 3000×) SEM images shown in Figure 1(f2–g2, f3–g3) justify that the HAp nanoparticles could disperse uniformly in the ST matrix of the porous HAp/ST composites. It implies that the HAp nanoparticles are well distributed and entrapped in the ST matrix, which induces the formation of rigid crystalline phases to retain the interconnected porous microstructures [20,22].

However, the lowest HAp nanoparticle content in the porous ST90 composite could lead to microstructural defects in terms of microstructure strength (structural collapse) and porosity (reduction). These are due to the insufficient interaction between the ST matrix and the HAp nanoparticles. The well dispersion of HAp nanoparticles in the ST matrix is due to the optimum HAp/ST proportion. The abundant intermolecular interactions between the well-distributed HAp nanoparticles and the sufficient ST matrix led to the formation of effective composite interfaces. It facilitates the formation of stable porous microstructures. The variations of the porous microstructures and the HAp/ST proportion impose a significant effect on the electrical properties of the porous HAp/ST composites.

**Figure 2:**
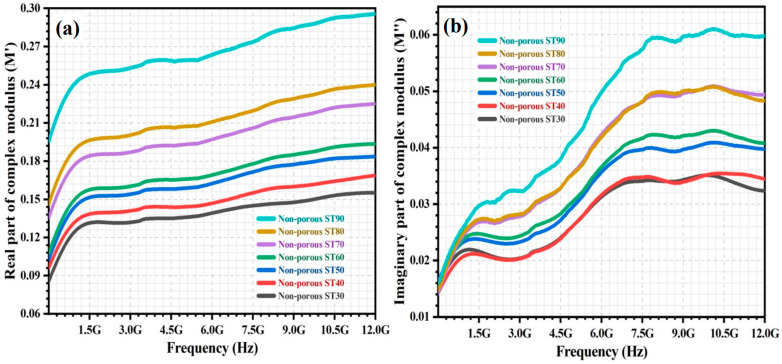
The variation of (**a**) M′ and (**b**) M″ of non-porous HAp/ST composites over frequency.

The complex modulus (M*) of the non-porous HAp/ST composites was investigated within a frequency range from 5 MHz to 12 GHz. Figure 2a,b show the frequency dependence of real modulus (M′) and imaginary modulus (M″) for the non-porous composites. The real modulus (M′) of all non-porous HAp/ST composites increases drastically in the lower frequency range and then increases gently with frequency. This might be due to the ease of polarons hopping mechanism in the low-frequency region. The charge carriers can move over long distances as the relaxation time at low frequencies is sufficient for alignment with the alteration of the electric field. It leads to the long-range mobility of charge carriers [10,16,23,24]. Generally, the M′ of the non-porous HAp/ST composites increases with frequency, which is attributed to the conduction phenomena due to the dominance of charge carriers with short-range mobility. It is associated with the insufficiency of restoring force to govern the mobility of charge carriers by exposure to a high-frequency electric field [14,18,25,26].

Meanwhile, the M″ of all the non-porous HAp/ST composites increases with frequencies. The M″ is the highest at high frequency. In Figure 2b, it can be found that the M″ of the non-porous HAp/ST composites gently increases when the frequency increases to 1.5 GHz. M″ increases drastically at high frequency. It levels off when the frequency is >8.0 GHz. It indicates the transition of the charge carrier from long-range to short-range mobility. The charge carriers are mobile over long distances in between the HAp/ST interfaces within a low-frequency range. It is due to the presence of charge carrier migration via long-range hopping from one site to the other [16,26,27]. The charge carriers are mobile within a short distance at a high frequency in between the HAp and ST phases due to the limitation of the potential well. It indicates the short-range localized motion of the charge carriers within the potential well [12,28,29]. It can be observed that the increment of ST content causes an increment of M′ and M″ of the non-porous HAp/ST composites as shown in Figure 2. This phenomenon might be due to the increment of polarization effects (electrode polarization and interfacial polarization) as well as the polar groups of ST in the HAp/ST composites when the ST proportion increases. It contributes to the mobility of charge carriers that are associated with the electrical transport phenomena [5,10,24].

**Figure 3:**
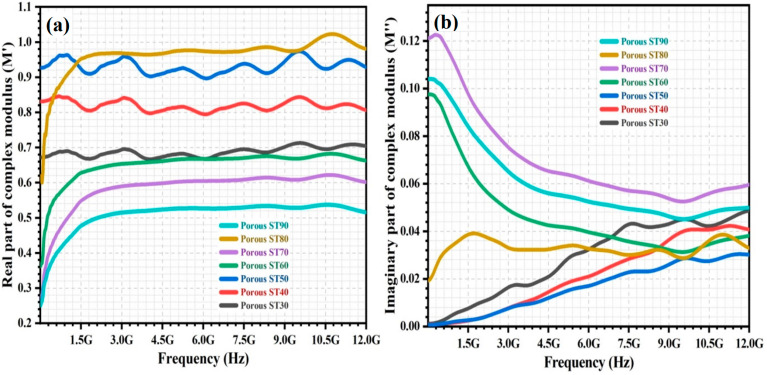
The variation of (**a**) M′ and (**b**) M″ of porous HAp/ST composites over frequency.

The variation of the M′ and M″ over frequency (5 MHz–12 GHz) for the porous HAp/ST composites is shown in Figure 3a,b, respectively. In Figure 3a, the multiple relaxation peaks could be observed in the M′ spectra of the porous HAp/ST composites with low ST proportion (30–50 wt%). In Figure 3b, M″ of the porous HAp/ST composites with low ST proportion (30–50 wt%) increases with frequency. The several step-like transitions in the complex modulus spectra of the porous HAp/ST composites (30–50 wt% ST proportion) indicate the presence of strong relaxation polarization processes. It might be attributed to the large agglomerated nanoparticles in the clustered porous microstructures. Both long-range and short-range mobility of the charge carriers is involved in the electrical transport process due to the imperfections within the material phases of the porous composites [1,4,30,31]. M′ and M″ of the porous HAp/ST composites increase and decrease, respectively, when the ST proportion increases from 30 wt% to 50 wt%. The small-size HAp agglomerates with large specific surface areas are more beneficial in increasing interface area compared with the large-size HAp agglomerates. As a result, the clustered porous microstructures with a low agglomeration effect are more favored in enhancing the electrical transport process [11,12,32].

The porous HAp/ST composites with high ST proportion (60–90 wt%) lead to a rapid increment of M′ for frequencies <1.5 GHz. M′ varies constantly for frequencies ≥ 1.5 GHz, as shown in Figure 3a. The M″ of the porous HAp/ST composites with high ST proportion 60–90 wt%) decreases gradually when frequency increases. The porous HAp/ST composites with high ST proportion (60–90 wt%) exhibit lower M′ and higher M″ than the porous HAp/ST composites with low ST proportion (30–50 wt%), as shown in Figure 3. This might be due to the effective electrostatic interactions between the dispersed HAp nanoparticles and polar groups of the ST matrix in the porous HAp/ST composites with a low agglomeration effect. The HAp/ST interactions hinder the polymer cooperative chain segmental dynamics and induce the accumulation of mobile charge carriers on the HAp/ST interfaces for the formation of a self-induced potential well. Hence, the short-range hopping of charge carriers is confined to the self-induced potential well [27,33,34,35]. As can be observed from Figure 3, M′ and M″ of the porous HAp/ST composites decrease and increase, respectively, when the ST proportion increases from 60 wt% to 90 wt%. As the starch proportion increases, the reduction of the agglomeration effect and the increment of interfacial interaction signify the high ionic conductivity [15,25].

In particular, the porous ST80 composite (80 wt% ST proportion) exhibits higher M′ than the other porous HAp/ST composites, as shown in Figure 3a. It can be observed that the M″ of the porous ST80 composite shows various variations over frequency. The M″ of the porous ST80 composite is the lowest among the porous HAp/ST composites with a high ST proportion ≥60 wt%. This might be due to the hierarchical porous microstructure of the porous HAp/ST composite with even distribution of HAp nanoparticles. In the meantime, the porosity of this porous HAp/ST composite is also the highest. Abundant interconnected air voids can increase and reduces the activation energy of the charge carrier hopping process and the effective conductive pathways of the charge carrier motions, respectively [16,36,37].

**Figure 4:**
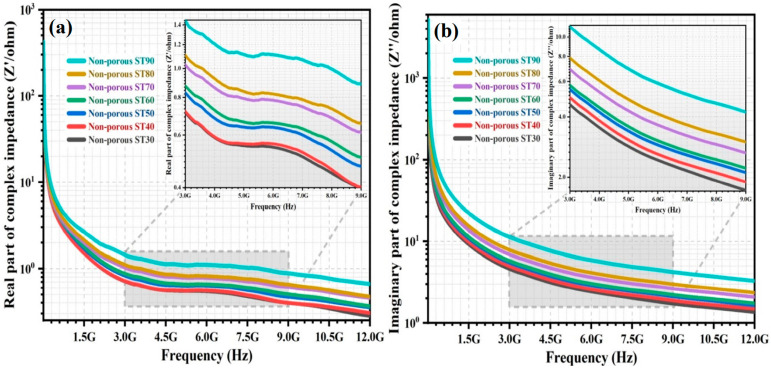
The variation of (**a**) Z′ and (**b**) Z″ of non-porous HAp/ST composites over frequency.

The complex impedance (Z*) of the non-porous HAp/ST composites was investigated within a frequency range from 5 MHz to 12 GHz. Figure 4a,b show the frequency dependence of real impedance (Z′) and imaginary impedance (Z″) of the non-porous composites.

Figure 4 demonstrates the frequency dependence of the Z* for the non-porous HAp/ST composites. Z′ and Z″ decrease as the frequency of the applied alternating electric field increases. This behaviour may be ascribed to the overall increment of the electrical conduction mechanism and the identical dielectric relaxation process for the non-porous composites. It is associated with the enhancement of hopping/tunneling transport of charge carriers between the localized ions when frequency increases [23,24,25,38]. The non-porous HAp/ST composites have maximal values for Z′ and Z” at low frequencies. It is because all charge carriers accumulate on the grain boundary interfaces. It leads to the formation of potential barriers against the charge carrier movement. The hopping of charge carriers is diminished due to the highly resistive grain boundaries at low frequencies. This is attributed to the agreement between Maxwell-Wagner interfacial polarization and Koop’s phenomenological theory [11,23,39,40]. At high frequency, Z′ and Z″ of the non-porous HAp/ST composites are small. It implies the possible release of charge carrier polarization/accumulation on the grain boundary interfaces and reduction of the energy barrier in the non-porous composites. Hence, the hopping of charge carriers within the crystalline grains contributes significantly to the electrical conductivity at high frequencies [12,31,41,42]. Z′ and Z″ of the non-porous HAp/ST composites increase when the ST proportion increases. It can be noticed in Figure 4. It corresponds to the enhancement of the resistive property of the non-porous composites and the reduction of effective charge carriers in the HAp nanoparticles that could impose a significant effect on electrical conduction [12,29,39].

**Figure 5:**
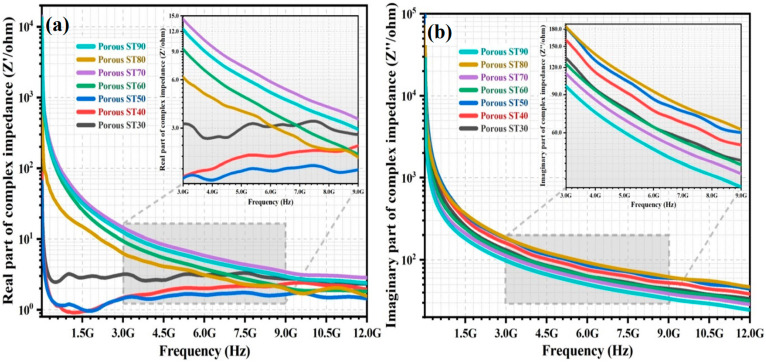
The variation of (**a**) Z′ and (**b**) Z″ of porous HAp/ST composites over frequency.

The variation of the Z′ and Z″ of the porous HAp/ST composites from 5 MHz to 12 GHz is shown in Figure 5a,b, respectively. The Z′ and Z″ of the porous HAp/ST composites decrease with frequency. The Z′ of the porous HAp/ST composites with low ST proportion (30–50 wt%) decreases drastically with increasing frequency. It remains constant at high frequency. In Figure 5a, it can also be observed that Z′ with ST proportion ≤ 50 wt% is lower than the porous HAp/ST composites with ST proportion ≥60 wt% due to the abundant charge carriers (enhancement of ac conductivity) from the contribution of HAp content [1,25]. It becomes significant, especially at low frequencies. At the high HAp content, the poor dispersion and agglomeration effect of the HAp nanoparticles are exhibited in the microstructures of the porous composites. The total grain boundary area of the agglomerated HAp nanoparticles is small for charge carrier accumulation. There are insufficient electrical barriers present in the grain boundary interfaces for the hopping of charge carriers [1,32,38]. In Figure 5a, it can be observed that the porous ST30 with the highest HAp content (70 wt%) exhibits higher Z′ and stronger relaxation phenomena than the other porous HAp/ST composites with high HAp content (≥50 wt%). It might be due to the presence of immobile charge carriers in the crystalline grain interiors of the highly agglomerated HAp nanoparticles [12,26,38].

In Figure 5, the Z′ and Z″ of the porous HAp/ST composites with high ST proportion (60–90 wt%) are higher and lower than the porous composites with low ST proportion (30–50 wt%), respectively. This can be ascribed to the highly insulative nature of the hierarchical porous microstructures. As the starch proportion increases from 60 wt% to 90 wt%, the insulative properties of the hierarchical porous microstructures are enhanced due to the increment of porosity. It reveals that the even distribution of air voids could maximize the Z′ of the porous HAp/ST composites. The interconnected pores of the hierarchical porous microstructures have smaller effective conducting volumes than the clustered porous microstructures as the pores show non-uniform and isolated distribution. The evenly distributed air voids tend to connect and form interconnected insulating channels to effectively block the continuous conductive pathway of the HAp/ST composites [32,41,43,44]. When the ST proportion is high, the reduction of the agglomeration effect enables the HAp nanoparticles to act as the micro-capacitors in the ST matrix and the accumulated charge carriers at the HAp/ST interfaces that could contribute to the polarization mechanisms (dipolar polarization and interfacial polarization). It induces a significant change in the reactance part of the dielectric system (Z″) for the porous composites [3,7,33,45].

However, the Z′ and Z″ of porous ST80 composite (80 wt% ST proportion) are lower and higher than other porous HAp/ST composites with high ST proportion (≥60 wt%), respectively, as shown in Figure 5. This might be due to the sufficient and homogenous HAp nanoparticles distribution in the robust hierarchical porous microstructures of the porous composite. Numerous tight interphases are formed between the ST matrix and the HAp nanoparticles with even distribution due to the large surface area of nanoparticles with minimum agglomeration effect. This effect leads to the reduction of ohmic resistivity on the interface of the heterogeneous systems [1,10,29,38]. The total grain boundary area of the HAp nanoparticles with even distribution is large for the charge carrier accumulation. It causes large electrical barriers present on the grain boundary interfaces. The strong electrostatic interactions between the HAp nanoparticles with even distribution and the polymer chain of the ST matrix produces results in a hindrance to the mobility of the polymer chain dipoles of ST. As a result, the Z″ of the porous ST80 composite is the highest [11,32,46].

**Figure 6:**
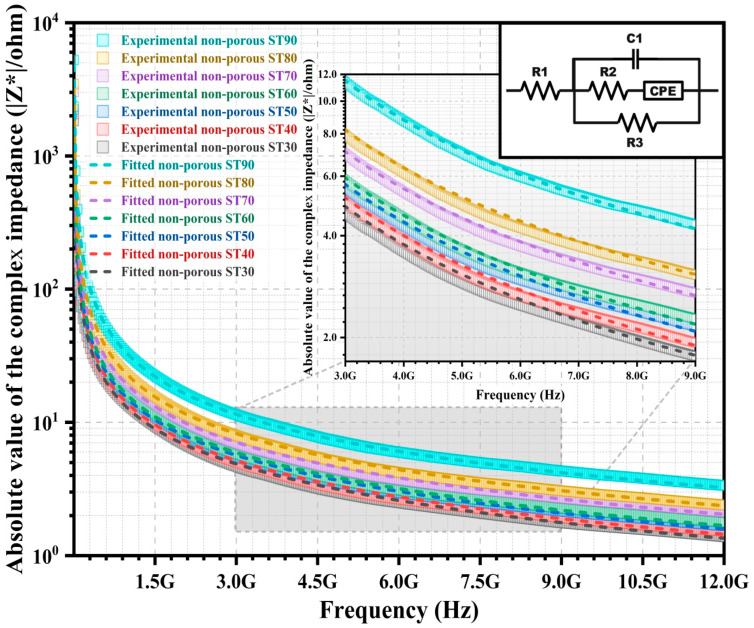
Impedance plot of non-porous HAp/ST composites with equivalent electrical circuit model (inset).

**Figure 7:**
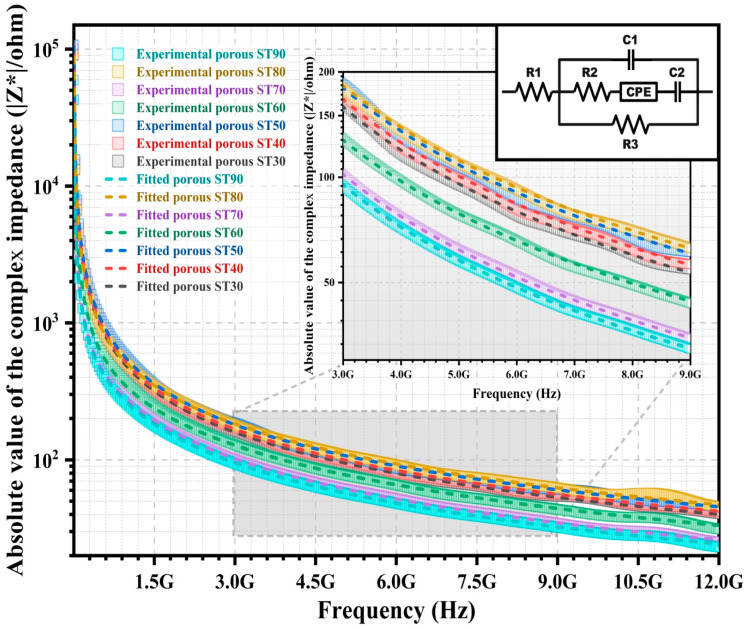
Impedance plot of porous HAp/ST composites with equivalent electrical circuit model (inset).

Impedance analysis via the development of an equivalent electrical circuit model is implemented for the correlation between microstructures and electrical behaviours of the HAp/ST composites. The |Z*| of the non-porous and porous HAp/ST composites with the equivalent electrical circuit models are shown in the inset of Figure 6 and Figure 7, respectively. Table 2 tabulates the electrical parameters of the equivalent electrical circuit models for the non-porous and porous HAp/ST composites. In Figure 6 and Figure 7, the chi-square (χ^2^) in Table 2 shows that the fitted |Z*| of each circuit model (dash lines) has good agreement with experimental results (open symbols) for the non-porous and porous composites. The best fit of the |Z*| spectra of the non-porous HAp/ST composites using an electrical equivalent circuit (Figure 6) consists of a capacitor (C1), a constant phase element (CPE), and three resistors (R1, R2, and R3). Meanwhile, the fitted |Z*| plots of the porous HAp/ST composites via measurement have good agreement with the equivalent electrical circuit (Figure 7). This circuit model consists of a constant phase element (CPE), two capacitors (C1 and C2), and three resistors (R1, R2, and R3). The serial resistor (R1) represents the intrinsic resistance. Parallel resistance (R3) refers to the charge transfer resistance of the composites. R1 (1.60–3.45 Ω) and R3 (1.25 × 10^5^–9.80 × 10^8^ Ω) of the porous HAp/ST composites are larger than the non-porous HAp/ST composites (R1: 0.22–0.46 Ω; R3: 0.75 × 10^4^–0.32 × 10^5^ Ω), as listed in Table 2. It suggests that the non-porous composites have a higher conductivity than the porous composites.

For the non-porous HAp/ST composites, the R1 and R3 increase as the ST proportion increases. It indicates the insulative behaviour of the ST. For the porous HAp/ST composites, high R1 and R3 are more remarkable in the hierarchical porous microstructures with high porosity. It implies the enhancement of the resistivity for the heterogeneous system. Parallel capacitor (C1) is attributed to the space charge region capacitance since the electrical charge carriers exist locally on the grain boundary of the composites. The C1 of the porous HAp/ST composites (27.67 × 10^−14^–54.66 × 10^−14^ F) is smaller than the non-porous HAp/ST composites (0.39 × 10^−11^–0.10 × 10^−10^ F), as shown in Table 2. It indicates that porosity is one of the crucial factors in determining the capacitive behaviour of the composites. The branch of series resistor (R2), constant phase element (CPE), and capacitor (C2) suggest the contributions of the complex electron processes to the interface regions (air/solid and solid/solid interfaces). In these equivalent electrical circuit models, the constant phase element (CPE) is used to account for the non-ideal capacitive response of the composite interfaces due to the heterogeneities of the microstructure and the material composition. The branch of the serial impedance elements of the porous HAp/ST composites (R2, CPE, and C2) exhibit a larger impedance than the non-porous HAp/ST composites (R2 and CPE). It is attributed to the application of high R2 values as well as the C2 element in the equivalent electrical circuit model of the porous composites. The three-dimensional porous microstructure of the composites is one of the conduction decisive factors, which is associated with electron transport efficiency.

## 4. Conclusions

In this study, the non-porous and porous HAp/ST composites with various HAp/ST proportions (70/30, 60/40, 50/50, 40/60, 30/70, 20/80, and 10/90 wt/wt%) were prepared. The effect of the ST proportion and the microstructural features on the electric behaviours of the HAp/ST composites was investigated. The reduction of the agglomeration effect and hierarchical porous microstructure formation of the composites are induced by the increment of the ST proportion. In modulus analysis, the high ST proportion of the composites enhances the mobility of charge carriers in the hopping processes. However, the highly porous hierarchal microstructure reduces the effective conductive pathways for the charge carrier motions. In impedance analysis, the low HAp proportion of the composites diminishes the effective charge carriers in the electrical conduction mechanism. The ST80 scaffold composite with high starch proportion (80 wt% ST proportion) and sufficient HAp content leads to the formation of a more significant porous microstructure with high interconnectivity (hierarchical porous morphology). Abundant interconnected air voids of the ST80 scaffold composite can increase and reduces the activation energy of the charge carrier hopping processes and the effective conductive pathways of the charge carrier motions, respectively. The highly porous hierarchal microstructure (interconnected porous microstructure with high porosity) of the ST80 scaffold composite enhances the overall impedance that is associated with electron transport efficiency. The electrical properties of non-porous and porous HAp/ST composites are reflected by electrical parameters of the equivalent electrical circuit models for indirect evaluation of the HAp/ST proportion and microstructure features. Thus, the electrical characterization has the potential to act as a gauging system for the determination of the material proportions and microstructural features of the porous ceramic/polymer-based composites.

## Figures and Tables

**Table 1:** Composition and porosity of the porous HAp/ST composites.

	Proportion (wt%)		
Composite	Hydroxyapatite	Starch	Porosity (%)
ST30	70	30	62.22 ± 0.43
ST40	60	40	63.96 ± 0.89
ST50	50	50	66.36 ± 0.78
ST60	40	60	64.72 ± 0.44
ST70	30	70	65.53 ± 0.67
ST80	20	80	66.56 ± 0.54
ST90	10	90	65.77 ± 0.33

**Table 2:** Electrical parameters of the equivalent circuit diagram for the non-porous and porous HAp/ST composites.

Composite	R1	R2	R3	C1	C2	CPE	χ2
	Ω			F		Fs^n−1^	
Non-porous ST30	0.26	22.8	0.75 × 10^4^	0.10 × 10^−10^	-	12.95 × 10^−11^	0.005
Non-porous ST40	0.28	22.0	0.16 × 10^5^	0.93 × 10^−11^	-	10.60 × 10^−11^	0.005
Non-porous ST50	0.28	22.0	0.20 × 10^5^	0.84 × 10^−11^	-	10.20 × 10^−11^	0.005
Non-porous ST60	0.33	22.0	0.21 × 10^5^	0.80 × 10^−11^	-	90.00 × 10^−12^	0.005
Non-porous ST70	0.37	21.0	0.22 × 10^5^	0.65 × 10^−11^	-	80.00 × 10^−12^	0.004
Non-porous ST80	0.40	19.0	0.25 × 10^5^	0.55 × 10^−11^	-	60.00 × 10^−12^	0.004
Non-porous ST90	0.44	19.0	0.32 × 10^5^	0.39 × 10^−11^	-	37.00 × 10^−12^	0.003
Porous ST30	2.15	19.5	9.80 × 10^8^	31.35 × 10^−14^	40.50 × 10^−15^	15.60 × 10^−13^	0.011
Porous ST40	2.20	19.5	9.50 × 10^8^	29.95 × 10^−14^	40.50 × 10^−15^	12.40 × 10^−13^	0.016
Porous ST50	2.30	19.5	9.50 × 10^8^	27.67 × 10^−14^	40.95 × 10^−15^	12.23 × 10^−13^	0.025
Porous ST60	1.60	10.6 × 10^2^	1.29 × 10^5^	39.80 × 10^−14^	21.00 × 10^−14^	13.30 × 10^−12^	0.008
Porous ST70	2.10	10.6 × 10^2^	1.30 × 10^5^	50.98 × 10^−14^	50.00 × 10^−14^	18.55 × 10^−12^	0.005
Porous ST80	3.45	22.0 × 10^2^	5.98 × 10^5^	28.20 × 10^−14^	87.00 × 10^−15^	91.00 × 10^−13^	0.006
Porous ST90	2.10	10.5 × 10^2^	1.25 × 10^5^	54.66 × 10^−14^	50.00 × 10^−14^	18.55 × 10^−12^	0.007

## Data Availability

The data presented in this study are available on request from the corresponding author. The data are not publicly available due to its confidentiality.
